# The controlling nutritional status score predicts postoperative mortality in patients with ruptured abdominal aortic aneurysm: a retrospective study

**DOI:** 10.3389/fcvm.2023.1129255

**Published:** 2023-04-27

**Authors:** Sheng-Lin Ye, Guang-Yuan Xiang, Zhao Liu, Wen-Dong Li, Tao Tang, Ai-Min Qian, Xiao-Qiang Li, Li-Li Sun

**Affiliations:** ^1^ Department of Vascular Surgery, Nanjing Drum Tower Hospital, The Affiliated Hospital of Nanjing University Medical School, Nanjing, China; ^2^ Department of Vascular Surgery, Nanjing Drum Tower Hospital Clinical College of Jiangsu University, Nanjing, China; ^3^ Department of Vascular Surgery, The Second Affiliated Hospital of Soochow University, Suzhou, China

**Keywords:** controlling nutritional status score, ruptured abdominal aortic aneurysm, prognosis, mid-term mortality, surgical complications

## Abstract

**Background:**

Ruptured abdominal aortic aneurysms (rAAAs) are challenging for vascular surgeons because they have a high mortality rate. In many diseases, nutritional status is closely associated with prognosis. The Controlling Nutritional Status (CONUT) screening tool score is a prognostic factor in some malignant and chronic diseases; however, the impact of nutritional status on rAAA has not yet been reported. In this study, we explored the relationship between the CONUT score and the postoperative prognosis of patients with rAAA.

**Methods:**

This was a retrospective review of 39 patients with rAAA who underwent surgical treatment from March 2018 to September 2021 at one center. Patient characteristics, nutritional status (CONUT score), and postoperative status were recorded. The patients were divided into groups A and B based on the CONUT score. The baseline characteristics of the two groups were compared, and Cox proportional hazards and logistic regression analyses were used to determine independent predictors of mid-term mortality and complications, respectively.

**Results:**

The overall mid-term mortality rate was 28.21% (11/39). Compared with group A, group B had higher intraoperative (*P* = 0.047) and mid-term mortality (*P* = 0.033) rates. The univariate analysis showed that age [hazard ratio (HR), 1.098; 95% confidence interval (CI), 1.019–1.182; *P* = 0.014], CONUT score (HR, 1.316; 95% CI, 1.027–1.686; *P* = 0.03), and surgical procedure (HR, 0.127; 95% CI, 0.016–0.992; *P* = 0.049) were associated with mid-term mortality, whereas the multivariate analysis showed that the CONUT score (HR, 1.313; 95% CI, 1.009–1.710; *P* = 0.043) was an independent predictor of mid-term mortality. The multivariate logistic regression analysis did not reveal any associations with complications. The Kaplan–Meier curves showed that group B had a lower mid-term survival rate (log-rank *P* = 0.024).

**Conclusion:**

Malnutrition is closely associated with the prognosis of patients with rAAA, and the CONUT score can be used to predict mid-term mortality.

## Introduction

Abdominal aortic aneurysm (AAA) is defined as an abdominal aorta diameter of >3 cm or ≥50% greater than the normal diameter as a result of irreversible pathological dilation (1). Ruptured AAA (rAAA), which is one of the most dangerous conditions in vascular surgery, has an extremely high mortality rate (1, 2) of up to 81% according to a recent report from the USA Preventive Services Task Force (3). The vast majority of deaths attributed to rupture occur before patients reach the operating room; however, the postoperative mortality rate still reportedly exceeds 40% (4). Some patients who reach the hospital alive forgo surgery because of the high cost, or they cannot undergo surgery because of the presence of serious comorbidities, including cardiovascular insufficiency.

AAA is a chronic degenerative disease of older individuals. Similarly, malnutrition is common in older patients with chronic diseases. In our clinical practice, we have observed that malnourished patients with rAAA have a high mortality rate. We therefore hypothesized that nutritional status is a prognostic factor in patients with rAAA.

The Controlling Nutritional Status (CONUT) tool for classifying nutritional status has attracted much attention recently (5). The CONUT score is a prognostic predictor in patients with some malignant or chronic diseases, such as end-stage liver disease (6) and acute heart failure (7). The CONUT score is also associated with disease activity in patients with lupus nephritis (8). In addition, the CONUT score is associated with prognosis and the treatment response in oncology (9–12). Most patients with rAAA have hypertension and are of an older age, which is consistent with the finding that a low CONUT score is directly associated with poor survival in older hospitalized patients with hypertension (13, 14).

The aim of this study was to evaluate the association between the CONUT score and prognosis, including death, in patients with rAAA. The CONUT score was calculated from preoperative laboratory test findings.

## Methods

### Study cohort

This was a single-center retrospective review of patients with rAAA. The study protocol conformed to the ethical guidelines of the Declaration of Helsinki and was approved by the Ethics Committee of Nanjing Drum Tower Hospital affiliated to Nanjing University School. All patients provided written informed consent for surgery.

**Figure 1:**
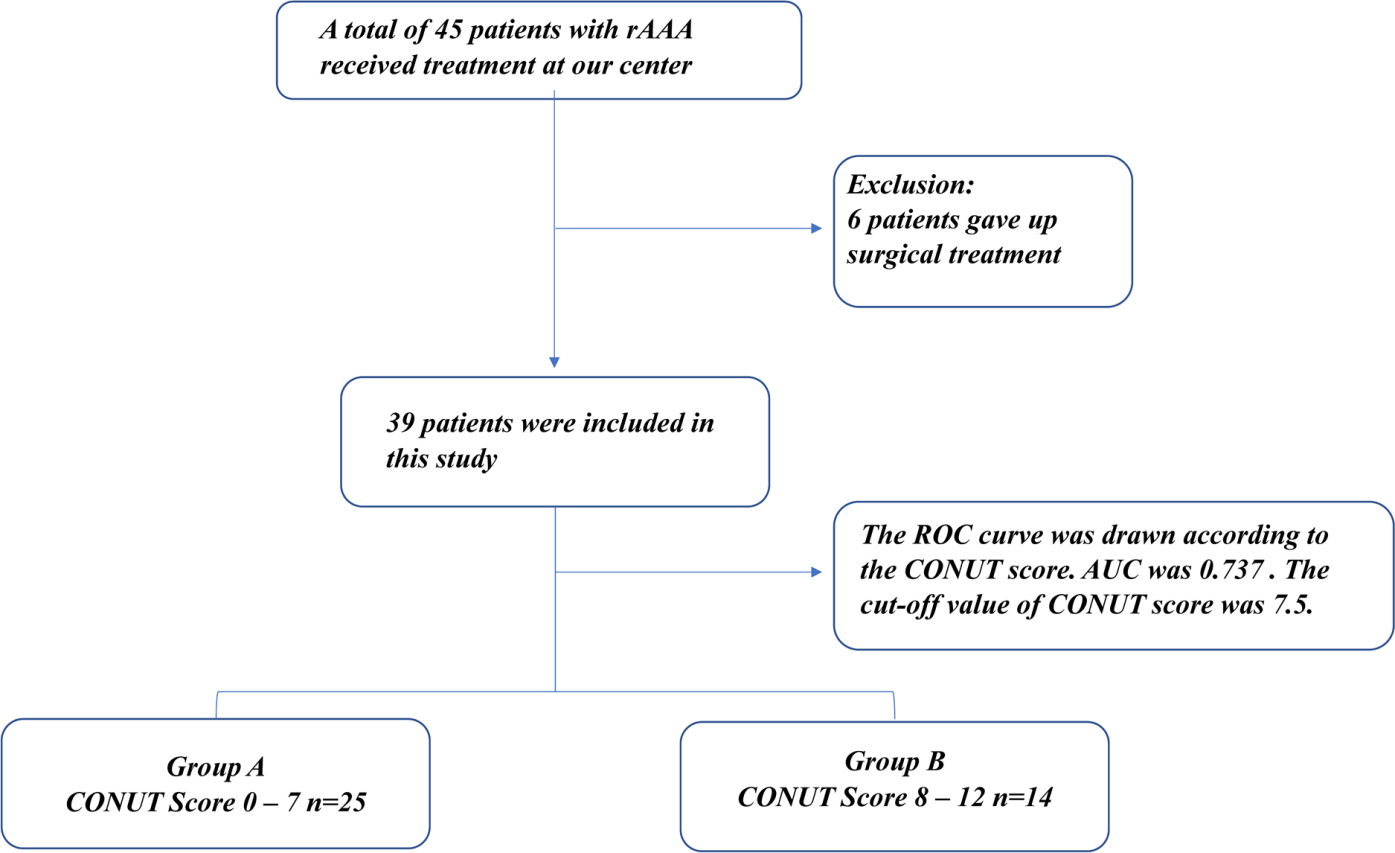
Flowchart of patients.

From March 2018 to September 2021, 45 patients with rAAA were admitted to our center as emergency cases. Six of the 45 patients were not managed surgically because of their poor physical condition. Endovascular aneurysm repair (EVAR) was performed in 22 patients, and open surgical repair (OSR) was performed in 17 patients. The 39 patients with rAAA were divided into two groups (Figure 1) according to the cut-off CONUT score: group A (CONUT score of 0–7, *n* = 25) and group B (CONUT score of 8–12, *n* = 14).

### Data collection and follow-up

We reviewed the clinical data of all patients, including their baseline characteristics (e.g., age, sex, comorbidities, smoking status, medication history); preoperative and postoperative laboratory findings (e.g., routine blood tests, coagulation function, liver and kidney function, postoperative B-type natriuretic peptide); surgical data (e.g., surgical method, surgical time, intraoperative blood transfusion, intraoperative blood loss); postoperative status (e.g., surgical complications, anesthesia recovery period, length of intensive care unit stay); and total hospitalization cost.

Postoperative follow-up mainly comprised regular physical examination and abdominal computed tomography. When the patient had stopped attending for follow-up, we contacted the patient or their family to determine their current status. Patients who were followed up at other institutions were contacted by telephone to obtain the required data.

### Clinical endpoints

The primary endpoint was mid-term mortality. The secondary endpoints were surgical complications, including acute organ injury, bleeding, and ischemia–reperfusion; implant-related complications, including stent rupture, leakage, implant infection, and vascular occlusion; and reoperation.

### Definitions

The CONUT scoring system was first proposed by de Ulibarri et al. in 2005 (5). The CONUT score is calculated by adding together the preoperative albumin concentration, lymphocyte count, and cholesterol concentration (Table 1). Patients were divided into four groups based on their CONUT scores. A CONUT score of 0–1 was classified as denoting a normal nutritional status, and CONUT scores of 2–4, 5–8, and 9–12 were classified as mild, medium, and severe malnutrition, respectively.

**Table 1:** Controlling nutritional status (CONUT) scores.

Parameter	Score			
Serum albumin, g/dl	≥3.5	3.0–3.49	2.50–2.99	<2.5
Albumin score	0	2	4	6
Total cholesterol, mg/dl	>180	140–180	100–139	<100
Cholesterol score	0	1	2	3
Lymphocytes, count/ml	≥1,600	1,200–1,599	800–1,199	<800
Lymphocyte score	0	1	2	3
Nutritional status score	0–1 (normal status)	2–4 (low risk)	5–8 (medium risk)	9–12 (severe risk)

### Statistical analysis

Normally distributed continuous variables are presented as the mean ± standard deviation. Non-normally distributed continuous variables are represented as the median [interquartile range (IQR)]. Categorical variables are presented as the number of patients (%). Independent and paired-samples *t*-tests, the Mann–Whitney *U* test, and analysis of variance were used for comparisons. The receiver operating characteristic (ROC) curve analysis was used to determine the cut-off value for the grouping. Survival rates were analyzed by the Kaplan–Meier method and the log-rank test. Correlations between patient characteristics and mortality were examined using Cox proportional hazards models. Surgical complications were analyzed by logistic regression. After the univariate analysis, any variable with a *P* value of <0.05 was entered into the multivariate analysis. All baseline characteristics, other studied variables, and comorbidities were incorporated into the Cox proportional hazards and logistic regression models to determine which factors were associated with mortality and postoperative complications. A *P* value of <0.05 was considered statistically significant. Data analysis was performed using SPSS 26.0 (IBM Corp., Armonk, NY, US).

## Results

### Patients' characteristics

**Figure 2:**
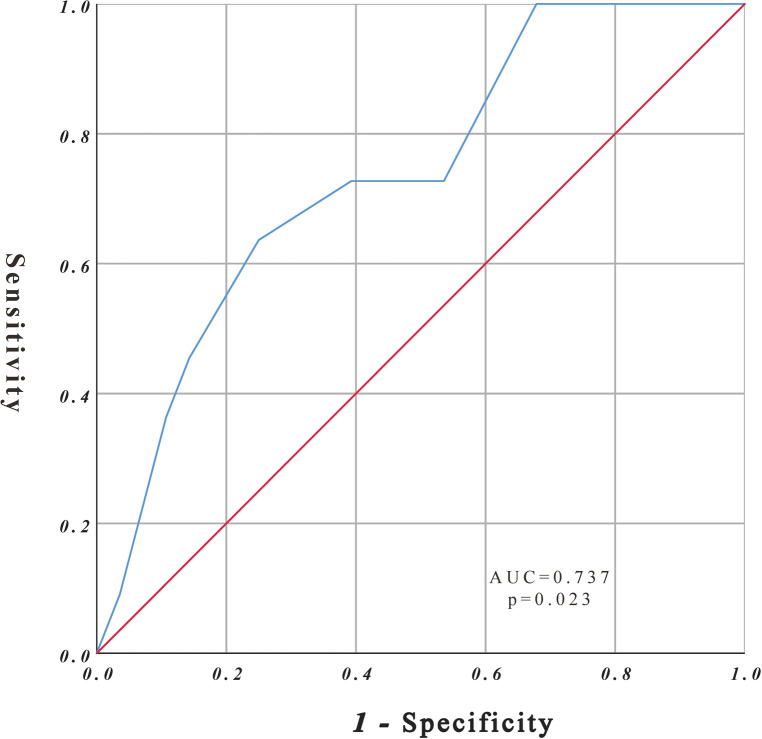
ROC curve analysis for survival rate.

The study cohort comprised 39 patients with rAAA. According to the CONUT score, all patients had varying degrees of malnutrition; 16 of 39 patients had mild malnutrition (41.0%), 15 had medium malnutrition (38.5%), and 8 had severe malnutrition (20.5%). Eleven patients died during follow-up, and the ROC curve was drawn according to the CONUT score to predict the time of death (Figure 2). The area under the ROC curve was 0.737 (95% CI, 0.568–0.906; *P* = 0.023), the cut-off CONUT score for determining the grouping was 7.5, the sensitivity was 0.636, and the specificity was 0.75. The study cohort comprised 39 patients with rAAA divided into two groups (Figure 1) according to the cut-off CONUT score: group A (CONUT score of 0–7, *n* = 25) and group B (CONUT score of 8–12, *n* = 14).

The patients' characteristics are shown in Table 2. The mean age of the patients in group A was 66.12 ± 11.52 years and in group B was 69.57 ± 7.30 years (*P* = 0.319). Most of the patients were male. Interestingly, the absence of women in group B, the group with a worse nutritional status, may indicate that older men have a poorer nutritional status than women, similar to the findings of a previous report (13). The median CONUT score was 4 (IQR, 3–6) in group A and 9.5 (IQR, 8–10) in group B (*P* < 0.001). Significant differences in serum albumin and total cholesterol were observed between the two groups (*P* < 0.001). In addition, the postoperative estimated glomerular filtration rate was significantly lower in group B than in group A (*P* = 0.009), indicating that postoperative renal function was worse in group B. This may account for the higher mortality rate in group B than in group A. Finally, patients in group B had a longer length of hospital stay than those in group A (*P* = 0.017) because patients with a poor nutritional status tended to take longer to adjust their physical function after surgery. No significant differences in other basic characteristics were observed between the two groups.

**Table 2:** Basic characteristics of included patients.

	Group A (*n *= 25)	Group B (*n *= 14)	*P*
*Age, years*	66.12 ± 11.52	69.57 ± 7.30	0.319
*Sex, male/female*	18/7	14/0	***0*** ***.*** ***036***
*AAA diameter, cm*	7.32 ± 3.38	7.52 ± 3.39	0.862
*Surgical method*			
EVAR	13 (52%)	5 (36%)	0.518
OSR	12 (48%)	9 (64%)	
*CONUT score*	4 (3–6)	9.5 (8–10)	***<0*** ***.*** ***001***
*Comorbid disease*			
Hypertension	21 (84%)	11 (79%)	0.686
DM	3 (12%)	0	0.54
Dyslipidemia	4 (16%)	1 (7%)	0.636
Stroke	1 (4%)	1 (7%)	1
Renal dysfunction	4 (16%)	3 (21%)	0.686
CAD	6 (24%)	3 (21%)	1
Prior arterial disease	5 (20%)	1 (7%)	0.391
Current smoker	16 (64%)	9 (64%)	1
*Medication*			
Depressor	17 (68%)	11 (79%)	0.713
Anticoagulants	11 (44%)	3 (21%)	0.187
*Laboratory examination*			
Serum albumin, mg/dl	34.15 ± 3.89	25.56 ± 2.57	***<0*** ***.*** ***001***
Total cholesterol, mg/dl	144.67 ± 31.62	105.93 ± 32.72	***0*** ***.*** ***001***
Lymphocyte count, 10^3^ ml	1.2 (0.65–1.65)	0.8 (0.8–0.95)	0.061
WBC, 10^9^/L	11.31 ± 5.38	10.45 ± 2.28	0.617
Hb, g/L	100.00 ± 21.79	98.36 ± 33.40	0.865
Plt, 109/L	122.00 ± 60.07	110.82 ± 77.81	0.648
CRP, mg/L	53.48 ± 40.53	71.97 ± 50.19	0.263
Postoperative eGFR, ml/min	79.10 ± 51.17	45.12 ± 19.62	***0*** ***.*** ***009***
Postoperative Cr, µmol/L	93.00 (55.10–194.90)	148.00 (117.00–230.70)	0.245
Postoperative PT, s	15.22 ± 6.52	14.05 ± 2.19	0.571
Postoperative Fibrinogen, g/L	2.48 ± 0.93	2.15 ± 0.92	0.341
Postoperative D-Dimer, mg/L	9.45 (3.67–21.06)	9.74 (6.38–13.37)	0.915
Postoperative BNP, pg/ml	79.00 (37.40–237.00)	70.65 (43.38–241.00)	0.959
*Preoperative situation*			
HR	85.92 ± 13.19	85.79 ± 11.81	0.975
SBP, mmHg	110.20 ± 26.97	106.21 ± 25.83	0.656
DBP, mmHg	66.56 ± 17.16	66.14 ± 16.70	0.942
*Intraoperative situation*			
Blood loss, ml	300 (150–2,675)	450 (100–4,250)	0.786
Blood transfusion, ml	1,650 (600–3,452)	2,822 (1,175–5,595)	0.335
Surgical time, h	3.10 ± 1.31	3.31 ± 1.79	0.665
*Postoperative situation*			
Anesthesia recovery period, h	16 (7–24)	42 (6.25–209.25)	0.07
ICU length of stay, d	2 (1–4.75)	6 (1.75–12.75)	***0*** ***.*** ***017***
*Length of stay, d*	12 (10–16)	14 (11–28)	0.24
*Cost,¥*	1,19,413.31 ± 59,434.46	1,58,214.32 ± 1,00,888.10	0.204

AAA, abdominal aortic aneurysm; EVAR, endovascular aneurysm repair; OSR, open surgical repair; CONUT, controlling nutritional status; DM, diabetes mellitus; CAD, coronary artery disease; WBC, white blood cell; Hb, hemoglobin; Plt, platelet; Cr, creatinine; PT, prothrombin time; INR, international standard ratio; eGFR, estimated glomerular filtration rate; BNP, type B natriuretic peptide; HR, heart rate; SBP, systolic blood pressure; DBP, diastolic blood pressure; ICU, intensive care unit. Continuous variables are presented as the mean ± standard deviation if normally distributed or the median (interquartile range) if not normally distributed. Categorical variables are presented as the number of patients (%).

The bold values indices are statistical difference between the two group.

### Complications and reoperation

Postoperative complications were identified in 19 patients, including 13 patients in group A and 6 patients in group B (Table 3). The difference was not statistically significant (*P* = 0.741). In group A, one patient developed an unexplained pulmonary vein embolism on postoperative day 8, four had acute postoperative renal insufficiency, two had pleural effusion caused by cardiac insufficiency, one had gastrointestinal bleeding caused by a stress ulcer accompanied by pulmonary ischemia–reperfusion injury on postoperative day 2, one had epilepsy of unknown cause on postoperative day 3, and one developed an incisional hernia in the sixth postoperative month. In addition, there was one case of type II stent leakage on postoperative day 9. There was also one case of type II stent leakage 3 months postoperatively and one case of stent rupture 1 month postoperatively, which was identified on postoperative computed tomography at follow-up, and both of these cases required reoperation. Moreover, one patient developed implant infection 6 months after surgery, which progressed to fatal sepsis. In group B, three patients were diagnosed as having Kidney Disease Improving Global Outcomes 3 acute postoperative kidney injury that improved with treatment, one had intestinal ischemic necrosis and pulmonary edema, one had a bleeding tendency that improved after emergency platelet transfusion, and two had different degrees of lung ischemia–reperfusion injury. Two of these patients required reoperation for implant-related or other vascular complications. According to the univariate logistic regression analysis, hypertension and long-term preoperative use of hypotensive agents were associated with complications or reoperation. However, according to the multivariate analysis, there were no significant independent associations (Table 4), which may be attributable to the small sample size.

**Table 3:** Patient's clinical end points.

	Group A (*n* = 25)	Group B (*n* = 14)	*P*
*Follow-up time, months*	17.16 ± 11.26	14.00 ± 13.69	0.442
*Surgical success*	24 (96%)	11 (79%)	0.123
*Intraoperative mortality*	1 (4%)	4 (29%)	***0*** ***.*** ***047***
*Midterm mortality*	4 (16%)	7 (50%)	***0*** ***.*** ***033***
*Reoperation*	5 (20%)	2 (14%)	1
*Total complications*	13 (52%)	6 (43%)	0.741
*Surgical complications*			
Acute organ injury	5 (20%)	3 (21%)	
Bleeding	1 (4%)	0	
Ischemia reperfusion	2 (8%)	2 (14%)	
Others	3 (12%)	2 (14%)	
*Implant-related complications*			
Stent rupture	1 (4%)	0	
Postoperative leakage	2 (8%)	0	
Implant infection	1 (4%)	0	
Vascular occlusion	0	1 (7%)	

The bold values indices are statistical difference between the two group.

**Table 4:** Logistic regression analysis of postoperative complications and reoperation.

Variable	Univariate analysis			Multivariate analysis		
	*P*	Odds ratio	95% CI	*P*	Odds ratio	95% CI
Age	0.482	0.977	0.916–1.042			
Sex	0.155	0.274	0.046–1.631			
CONUT score	0.835	1.025	0.813–1.291			
Surgical method	0.921	1.067	0.3–3.796			
AAA diameter	0.679	0.96	0.890–1.166			
Hypertension	0.043	10	1.070–93.437	0.224	6	0.335–107.420
DM	0.999					
Dyslipidemia	0.511	0.526	0.078–3.565			
Strok	0.999					
Renal dysfunction	0.313	2.5	0.422–14.828			
CAD	0.521	0.612	0.136–2.742			
Prior arterial disease	0.837	0.833	0.146–4.752			
Current smoker	0.306	2	0.531–7.539			
HR	0.346	0.975	0.925–1.028			
SBP	0.569	1.007	0.983–1.032			
DBP	0.704	1.007	0.970–1.047			
Hypotensor	0.045	4.8	1.034–22.293	0.585	1.8	0.219–14.801
Anticoagulants	0.719	0.786	0.212–2.918			
WBC	0.351	0.924	0.782–1.091			
Hb	0.573	0.992	0.966–1.020			
Plt	0.71	1.002	0.992–1.013			

AAA, abdominal aortic aneurysm; CONUT, controlling nutritional status; DM, diabetes mellitus; CAD, coronary artery disease; WBC, white blood cell; Hb, hemoglobin; Plt, platelet; Cr, creatinine; HR, heart rate; SBP, systolic blood pressure; DBP, diastolic blood pressure.

### Intraoperative mortality

Five patients died during surgery (12.82%), and the analysis revealed a significant difference between group A and B (*P* = 0.047). Four patients in group B died of persistent hypotension that could not be resolved by blood transfusion and fluid rehydration. One patient in group A demonstrated iliac artery occlusion intraoperatively, prompting the surgeon to consider OSR. However, when informed of the situation, his family decided to cease active treatment.

### Mid-term mortality

**Figure 3:**
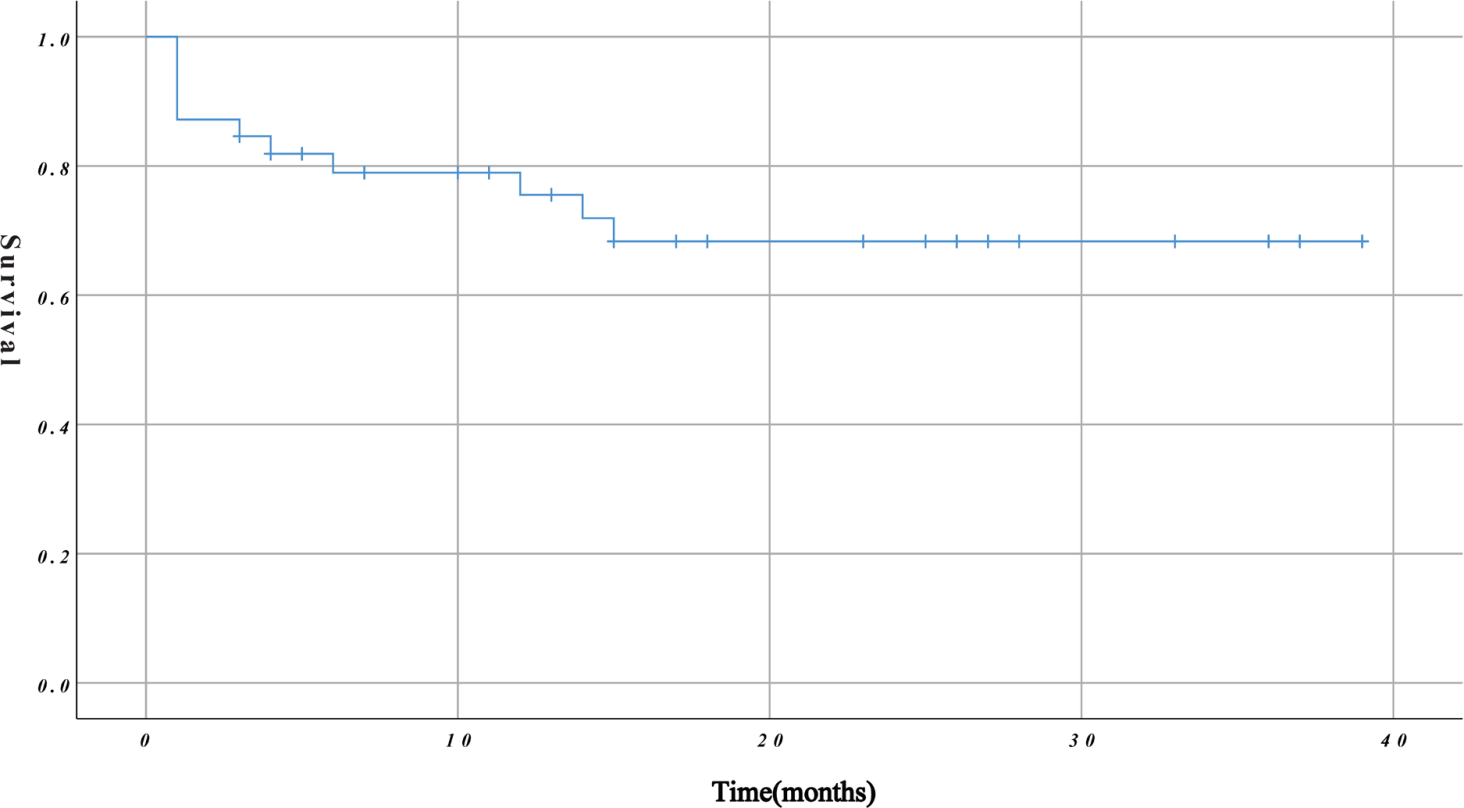
Kaplan–Meier curves for overall survival rate.

The mean duration of follow-up was 16.03 ± 12.11 months. The overall survival rate during follow-up was 71.79% (79.49% and 76.92% at 6 and 12 months, respectively) (Figure 3). The mean duration of follow-up in group A and group B was 17.16 ± 11.26 months and 14.00 ± 13.69 months, respectively. The difference between the two groups was not statistically significant (*P* = 0.422). However, on further analysis, we found that group B had a higher mid-term mortality rate than group A (*P* = 0.033).

**Figure 4:**
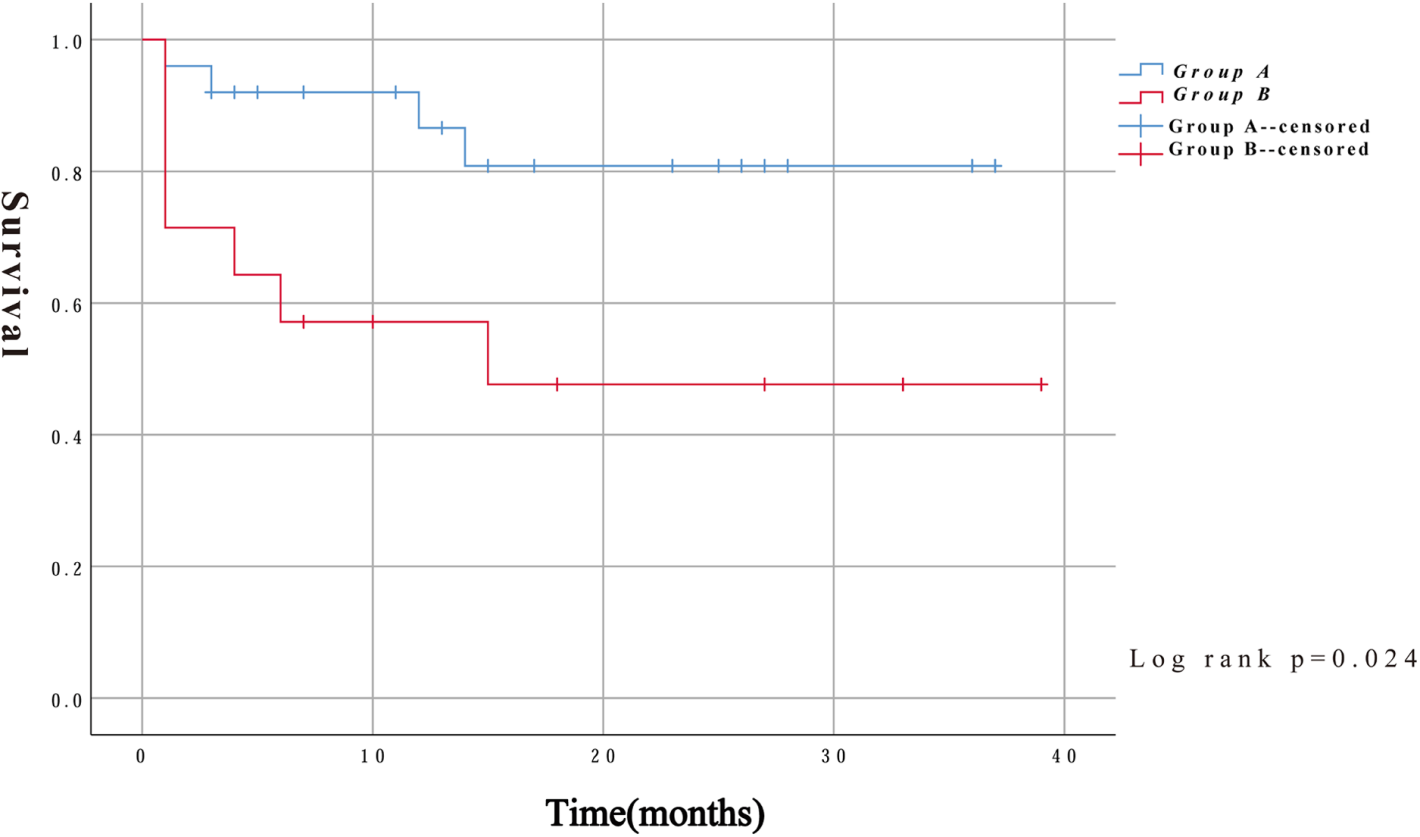
Kaplan Meier curves for midterm survival were compared among the two groups.

In addition to intraoperative deaths, two patients in group A died of aneurysm rupture and another died of severe lung infection 14 months after surgery. Three patients in group B died at 4, 14, and 15 months after surgery for multiple-organ failure, severe pulmonary infection, and exacerbation of renal failure, respectively. Kaplan–Meier survival curves were constructed using the follow-up data. As shown in Figure 4, the survival rates were significantly lower in group B than in group A (log-rank, *P* = 0.024).

We used the Cox proportional hazards model to predict risk factors for mortality. The univariate analysis showed that age (HR, 1.098; 95% CI, 1.019–1.182; *P* = 0.014), CONUT score (HR, 1.316; 95% CI, 1.027–1.686; *P* = 0.03), and surgical procedure (HR, 0.127; 95% CI, 0.016–0.992; *P* = 0.049) were risk factors for mortality. The multivariate analysis using these three factors showed that the CONUT score (HR, 1.313; 95% CI, 1.009–1.710; *P* = 0.043) was an independent risk factor for mortality in patients with rAAA (Table 5).

**Table 5:** Cox regression analysis for risks of midterm mortality.

Variable	Univariate analysis			Multivariate analysis		
	*P*	Hazard ratio	95% CI	*P*	Hazard ratio	95% CI
Age	0.014	1.098	1.019–1.182	0.083	1.074	0.991–1.165
Sex	0.308	0.035	0.000–21.768			
CONUT score	0.03	1.316	1.027–1.686	0.043	1.313	1.009–1.710
Surgical method	0.049	0.127	0.016–0.992	0.098	0.17	0.021–1.389
AAA diameter	0.135	0.949	0.684–1.052			
Hypertension	0.938	1.065	0.230–4.944			
DM	0.495	23.354	0.003–1,97,532.254			
Dyslipidemia	0.369	26.217	0.021–32,557.226			
Strok	0.748	0.713	0.091–5.609			
Renal dysfunction	0.975	1.024	0.221–4.744			
CAD	0.637	1.447	0.312–6.700			
Prior arterial disease	0.944	0.946	0.204–4.394			
Current smoker	0.106	0.279	0.059–1.312			
HR	0.253	0.968	0.916–1.023			
SBP	0.297	1.012	0.989–1.036			
DBP	0.294	1.02	0.983–1.058			
Hypotensor	0.424	0.535	0.116–2.480			
Anticoagulants/Antiplatelet	0.86	1.117	0.326–3.826			
WBC	0.659	1.036	0.886–1.212			
Hb	0.066	0.969	0.936–1.002			
Plt	0.596	1.003	0.991–1.015			

AAA, abdominal aortic aneurysm; CONUT, controlling nutritional status; DM, diabetes mellitus; CAD, coronary artery disease; WBC, white blood cell; Hb, hemoglobin; Plt, platelet; Cr, creatinine; HR, heart rate; SBP, systolic blood pressure; DBP, diastolic blood pressure.

## Discussion

rAAAs are often lethal, with most deaths occurring because the patient does not make it to the operating room. Lindholt et al. found that the mortality rate of patients without surgical intervention could reach 100% (15). The latest Society for Vascular Surgery guidelines indicate that patients with rAAA require immediate emergency surgery, and the window for successful intervention is no more than 90 min (16, 17). Despite surgical treatment, the inpatient mortality rate is still as high as 40% (4). Our findings are consistent with these data. Six of the 45 patients who were admitted to our center for rAAA from March 2018 to September 2021 were unable to undergo surgical treatment because of their poor physical condition or financial factors. The remaining 39 patients underwent emergency surgery, and the postoperative mortality rate of these patients was 28.21% (11/39).

Previous attempts to identify prognostic factors in patients with rAAA have focused on surgical procedures. Several studies have shown that the annual decline in morbidity with rAAA in Europe and the United States over the past 20 years is closely related to the increasing proportion of patients undergoing EVAR (18, 19). Several randomized controlled trials have shown EVAR to be significantly superior to OSR in terms of early survival; however, there is no statistically significant difference in long-term survival between these procedures (20–22). Of note, both of these studies included patients with AAA, whether ruptured or unruptured. However, for rAAA alone, three recent large randomized controlled trials have found no clear evidence that EVAR is superior to OSR in terms of early survival (23–25). Interestingly, in the present study, we found that mid-term mortality of patients with rAAA was related to the surgical method (*P* = 0.049). Specifically, patients who underwent OSR had higher postoperative mortality than those who underwent EVAR. However, these results may be directly related to the small sample size or to subjective biases in the surgeons' choices of procedure.

In this retrospective analysis, we first proposed a correlation between nutritional status and the prognosis of patients with rAAA. Nutritional status is a good indicator of both a patient's overall general condition and their immune and metabolic capacity. The CONUT screening tool score has been shown to predict the outcomes of malignant, chronic, and cardiovascular diseases (6, 7, 13, 14, 26). In this study, we found that 58.97% of patients with rAAA (23/39) had moderate or severe malnutrition, which may have been caused by prior massive bleeding from rAAA. The mid-term mortality of these patients was as high as 34.78% (8/23), which was much higher than that of patients who were at a normal or low risk (18.75%, 3/16). The univariate and multivariate analyses to identify predictors of mortality from any cause found that the CONUT score was an independent predictor of mid-term mortality (HR, 1.313; *P* = 0.043), suggesting that nutritional status influences the outcomes of patients undergoing surgical treatment for rAAA. Moreover, the logistic regression analysis showed that a high CONUT score was not associated with postoperative complications. The findings of previous studies investigating the correlations between the CONUT score and postoperative complications have been conflicting. Kodama et al. reported that the CONUT score predicts not only overall survival after OSR in patients with AAA, but also postoperative complications (27). Interestingly, in their study of radical hepatectomy for intrahepatic cholangiocarcinoma, Miyata et al. (28) found that a high CONUT score was associated with poorer postoperative overall survival, but not with postoperative complications, which is consistent with our findings.

Possible explanations for the high mortality rate among malnourished rAAA patients include the following. Malnutrition is often closely associated with frailty, which is defined as a clinically identifiable state of increased vulnerability and dysfunction (29, 30). Additionally, nutritional status partly reflects the development of inflammation (31–33), which promotes cytokine production and muscle catabolism, suppresses appetite, and lowers the albumin concentration (34). Reduced albumin may increase blood viscosity and activate platelets, leading to a deterioration in endothelial function (35). Furthermore, a previous study showed a relationship between nutritional status and C-reactive protein and interleukin-6 concentrations in humans (36). Moreover, the maximum diameter of AAA is positively correlated with the concentrations of interleukin-6, C-reactive protein, and other inflammatory factors (37). Cytokines secreted by inflammatory cells can damage tissues, causing the walls of blood vessels to become less elastic and eventually rupture (38). There have been no specific reports on the prognostic value of serum total cholesterol in cardiovascular disease, but low cholesterol is associated with a poor prognosis in a variety of cancers (39, 40). Therefore, it could be speculated that patients with a low total cholesterol concentration have a worse underlying condition and more comorbidities.

To the best of our knowledge, this is the first study to propose that nutritional status plays an important role in the prognosis of patients with rAAA, and that the CONUT score can predict mid-term mortality. In this study, malnutrition was common in patients with rAAA, and as malnutrition became more severe, mid-term mortality increased. Therefore, we suggest that clinicians should integrate the recognition of malnutrition into their daily practice and focus on nutritional health education for patients with AAA with the aim of reducing mortality.

This study has some limitations that should be noted. First, it was a single-center retrospective study. Furthermore, because most patients with rAAA die on the way to hospital, the study cohort was small. Second, updating our hospital's medical record system resulted in loss of case data from before March 2018. Additionally, because most of the patients were transferred from primary hospitals, we lacked some preoperative laboratory tests, despite attempting to collect as much information as possible through telephone follow-up. Therefore, we had no choice but to abandon some aspects of the preoperative examination and focus on postoperative data. Third, there was selection bias in the procedures performed, which were chosen mainly based on the personal judgment of the surgeon. Thus, we could not validly investigate the relationship between the surgical procedure and the prognosis of the patients. In addition, because of the rapid onset and urgency of rAAA, we lacked detailed preoperative imaging findings concerning the anatomical features of the aorta in some patients. Fourth, follow-up was limited; therefore, further studies are needed to understand the impact of nutritional status on long-term clinical outcomes. Finally, we did not compare the prognostic value of the scores obtained from other nutritional screening tools in these patients; however, a previous study showed that the CONUT score has good predictive performance in patients with AAA (27). To validate the effect of nutritional status on patient prognosis, further investigation in different clinical settings will be necessary. Therefore, we advocate that future studies should examine the potential role of nutritional status assessment. Further research is also needed to determine whether malnourished patients benefit from nutritional supplements.

## Conclusion

rAAA has a high mortality rate, and nutritional status is associated with mid-term mortality. The score of the new nutritional screening tool, CONUT, is easy to determine in clinical practice. Based on our study, the CONUT score may play a prognostic role in rAAA. Clinicians should focus on patients' nutritional status and educate patients about good nutritional practices to improve their outcomes. Future large multi-center studies are needed to confirm our findings.

## Data Availability

The original contributions presented in the study are included in the article/Supplementary Material, further inquiries can be directed to the corresponding authors.

## References

[1] Wanhainen A Verzini F Van Herzeele I Allaire E Bown M Cohnert T Editor's choice - European society for vascular surgery (ESVS) 2019 clinical practice guidelines on the management of abdominal aorto-iliac artery aneurysms. Eur J Vasc Endovasc Surg. (2019) 57(1):8–93. 10.1016/j.ejvs.2018.09.020 30528142

[2] Lesperance K Andersen C Singh N Starnes B Martin MJ. Expanding use of emergency endovascular repair for ruptured abdominal aortic aneurysms: disparities in outcomes from a nationwide perspective. J Vasc Surg. (2008) 47(6):1165. 10.1016/j.jvs.2008.01.055 18394857

[3] US Preventive Services Task Force, Owens DK Davidson KW Krist AH Barry MJ Cabana M Screening for abdominal aortic aneurysm: US preventive services task force recommendation statement. J Am Med Assoc. (2019) 322(22):2211–8. 10.1001/jama.2019.18928 31821437

[4] Karthikesalingam A Holt PJ Vidal-Diez A Ozdemir BA Poloniecki JD Hinchliffe RJ Mortality from ruptured abdominal aortic aneurysms: clinical lessons from a comparison of outcomes in England and the USA. Lancet. (2014) 383(9921):963–9. 10.1016/S0140-6736(14)60109-4 24629298

[5] Ignacio de Ulibarri J Gonzalez-Madrono A de Villar NG Gonzalez P Gonzalez B Mancha A CONUT: a tool for controlling nutritional status. First validation in a hospital population. Nutr Hosp. (2005) 20(1):38–45. 15762418

[6] Fukushima K Ueno Y Kawagishi N Kondo Y Inoue J Kakazu E The nutritional index ‘CONUT’ is useful for predicting long-term prognosis of patients with end-stage liver diseases. Tohoku J Exp Med. (2011) 224(3):215–9. 10.1620/tjem.224.215 21701127

[7] Shirakabe A Hata N Kobayashi N Okazaki H Matsushita M Shibata Y The prognostic impact of malnutrition in patients with severely decompensated acute heart failure, as assessed using the prognostic nutritional index (PNI) and controlling nutritional status (CONUT) score. Heart Vessels. (2018) 33(2):134–44. 10.1007/s00380-017-1034-z 28803356

[8] Ahn SS Yoo J Jung SM Song JJ Park YB Lee SW. Comparison of the clinical implications among five different nutritional indices in patients with lupus nephritis. Nutrients. (2019) 11(7):1456. 10.3390/nu11071456 PMC6682980 31252552

[9] Takagi K Buettner S Ijzermans JNM. Prognostic significance of the controlling nutritional status (CONUT) score in patients with colorectal cancer: a systematic review and meta-analysis. Int J Surg. (2020) 78:91–6. 10.1016/j.ijsu.2020.04.046 32335238

[10] Terasaki F Sugiura T Okamura Y Ito T Yamamoto Y Ashida R The preoperative controlling nutritional status (CONUT) score is an independent prognostic marker for pancreatic ductal adenocarcinoma. Updates Surg. (2021) 73(1):251–9. 10.1007/s13304-020-00792-9 32410163

[11] Zhang Y Zhang X. Controlling nutritional status score, a promising prognostic marker in patients with gastrointestinal cancers after surgery: a systematic review and meta-analysis. Int J Surg. (2018) 55:39–45. 10.1016/j.ijsu.2018.05.018 29783000

[12] Shimose S Kawaguchi T Iwamoto H Tanaka M Miyazaki K Ono M Controlling nutritional status (CONUT) score is associated with overall survival in patients with unresectable hepatocellular carcinoma treated with lenvatinib: a multicenter cohort study. Nutrients. (2020) 12(4):1076. 10.3390/nu12041076 32295043 PMC7231015

[13] CabreMFerreiroCArusMRocaMPalomeraESerra-PratM. Evaluation of CONUT for clinical malnutrition detection and short-term prognostic assessment in hospitalized elderly people. J Nutr Health Aging. (2015) 19(7):729–33. 10.1007/s12603-015-0536-626193855

[14] Sun XN Luo LM Zhao XQ Ye P. Controlling nutritional status (CONUT) score as a predictor of all-cause mortality in elderly hypertensive patients: a prospective follow-up study. BMJ Open. (2017) 7(9):e015649. 10.1136/bmjopen-2016-015649 PMC5623525 28928176

[15] Lindholt JS Sogaard R Laustsen J. Prognosis of ruptured abdominal aortic aneurysms in Denmark from 1994 to 2008. Clin Epidemiol. (2012) 4:111–3. 10.2147/CLEP.S31098 22701090 PMC3372968

[16] Rokosh RS Wu WW Eskandari MK Chaikof EL. Society for vascular surgery implementation of guidelines in abdominal aortic aneurysms: preoperative surveillance and threshold for repair. J Vasc Surg. (2021) 74(4):1053–4. 10.1016/j.jvs.2021.04.068 34022377

[17] Chaikof EL Dalman RL Eskandari MK Jackson BM Lee WA Mansour MA The society for vascular surgery practice guidelines on the care of patients with an abdominal aortic aneurysm. J Vasc Surg. (2018) 67(1):2–77 e2. 10.1016/j.jvs.2017.10.044 29268916

[18] Reite A Soreide K Ellingsen CL Kvaloy JT Vetrhus M. Epidemiology of ruptured abdominal aortic aneurysms in a well-defined Norwegian population with trends in incidence, intervention rate, and mortality. J Vasc Surg. (2015) 61(5):1168–74. 10.1016/j.jvs.2014.12.054 25659456

[19] Parkinson F Ferguson S Lewis P Williams IM Twine CP Rupture rates of untreated large abdominal aortic aneurysms in patients unfit for elective repair. J Vasc Surg. (2015) 61(6):1606–12. 10.1016/j.jvs.2014.10.023 25661721

[20] United Kingdom EVAR Trial Investigators, Greenhalgh RM Brown LC Powell JT Thompson SG Epstein D Endovascular versus open repair of abdominal aortic aneurysm. N Engl J Med. (2010) 362(20):1863–71. 10.1056/NEJMoa0909305 20382983

[21] Lederle FA Freischlag JA Kyriakides TC Padberg FT Jr Matsumura JS Kohler TR Outcomes following endovascular vs open repair of abdominal aortic aneurysm: a randomized trial. J Am Med Assoc. (2009) 302(14):1535–42. 10.1001/jama.2009.1426 19826022

[22] Patel R Sweeting MJ Powell JT Greenhalgh RM Investigators ET. Endovascular versus open repair of abdominal aortic aneurysm in 15-years’ follow-up of the UK endovascular aneurysm repair trial 1 (EVAR trial 1): a randomised controlled trial. Lancet. (2016) 388(10058):2366–74. 10.1016/S0140-6736(16)31135-7 27743617

[23] Desgranges P Kobeiter H Katsahian S Bouffi M Gouny P Favre JP Editor's choice - ECAR (endovasculaire ou chirurgie dans les anevrysmes aorto-iliaques rompus): a French randomized controlled trial of endovascular versus open surgical repair of ruptured aorto-iliac aneurysms. Eur J Vasc Endovasc. (2015) 50(3):303–10. 10.1016/j.ejvs.2015.03.028 26001320

[24] Powell JT Sweeting MJ Thompson MM Ashleigh R Bell R Gomes M Endovascular or open repair strategy for ruptured abdominal aortic aneurysm: 30 day outcomes from IMPROVE randomised trial. Brit Med J. (2014) 348:f661. 10.1136/bmj.f7661 24418950

[25] Kapma MR Dijksman LM Reimerink JJ de Groof AJ Zeebregts CJ Wisselink W Cost-effectiveness and cost-utility of endovascular versus open repair of ruptured abdominal aortic aneurysm in the Amsterdam acute aneurysm trial. Brit J Surg. (2014) 101(3):208–15. 10.1002/bjs.9356 24469619

[26] Kunimura A Ishii H Uetani T Aoki T Harada K Hirayama K Impact of nutritional assessment and body mass index on cardiovascular outcomes in patients with stable coronary artery disease. Int J Cardiol. (2017) 230:653–8. 10.1016/j.ijcard.2017.01.008 28077227

[27] Kodama A Takahashi N Sugimoto M Niimi K Banno H Komori K. Associations of nutritional status and muscle size with mortality after open aortic aneurysm repair. J Vasc Surg. (2019) 70(5):1585–93. 10.1016/j.jvs.2019.01.049 30898367

[28] Miyata T Yamashita YI Higashi T Taki K Izumi D Kosumi K The prognostic impact of controlling nutritional status (CONUT) in intrahepatic cholangiocarcinoma following curative hepatectomy: a retrospective single institution study. World J Surg. (2018) 42(4):1085–91. 10.1007/s00268-017-4214-1 28887635

[29] Xue QL. The frailty syndrome: definition and natural history. Clin Geriatr Med. (2011) 27(1):1. 10.1016/j.cger.2010.08.009 21093718 PMC3028599

[30] Fried LP Tangen CM Walston J Newman AB Hirsch C Gottdiener J Frailty in older adults: evidence for a phenotype. J Gerontol A Biol Sci Med Sci. (2001) 56(3):M146–56. 10.1093/gerona/56.3.M146 11253156

[31] Raiten DJ Ashour FAS Ross AC Meydani SN Dawson HD Stephensen CB Inflammation and nutritional science for programs/policies and interpretation of research evidence (INSPIRE). J Nutr. (2015) 145(5):1039s–108s. 10.3945/jn.114.194571 25833893 PMC4448820

[32] Zabetakis I Lordan R Norton C Tsoupras A. COVID-19: the inflammation link and the role of nutrition in potential mitigation. Nutrients. (2020) 12(5):1466. 10.3390/nu12051466 PMC7284818 32438620

[33] Alwarawrah Y Kiernan K MacIver NJ. Changes in nutritional status impact immune cell metabolism and function. Front Immunol. (2018) 9:1055. 10.3389/fimmu.2018.01055 29868016 PMC5968375

[34] Merker M Felder M Gueissaz L Bolliger R Tribolet P Kagi-Braun N Association of baseline inflammation with effectiveness of nutritional support among patients with disease-related malnutrition A secondary analysis of a randomized clinical trial. JAMA Netw Open. (2020) 3(3):e200663. 10.1001/jamanetworkopen.2020.0663 32154887 PMC7064875

[35] Joles JA WillekesKoolschijn N Koomans HA. Hypoalbuminemia causes high blood viscosity by increasing red cell lysophosphatidylcholine. Kidney Int. (1997) 52(3):761–70. 10.1038/ki.1997.393 9291198

[36] Smidowicz A Regula J. Effect of nutritional status and dietary patterns on human serum C-reactive protein and interleukin-6 concentrations. Adv Nutr. (2015) 6(6):738–47. 10.3945/an.115.009415 26567198 PMC4642421

[37] Diehm N Dick F Schaffner T Schmidli J Kalka C Di Santo S Novel insight into the pathobiology of abdominal aortic aneurysm and potential future treatment concepts. Prog Cardiovasc Dis. (2007) 50(3):209–17. 10.1016/j.pcad.2007.05.001 17976505

[38] Gitlin JM Trivedi DB Langenbach R Loftin CD. Genetic deficiency of cyclooxygenase-2 attenuates abdominal aortic aneurysm formation in mice. Cardiovasc Res. (2007) 73(1):227–36. 10.1016/j.cardiores.2006.10.015 17137566

[39] Li JR Zhang Y Zheng JL. Decreased pretreatment serum cholesterol level is related with poor prognosis in resectable non-small cell lung cancer. Int J Clin Exp Patho. (2015) 8(9):11877–83. PMC4637759 26617943

[40] Shin HJ Roh CK Son SY Hoon H Han SU. Prognostic value of hypocholesterolemia in patients with gastric cancer. Asian J Surg. (2021) 44(1):72–9. 10.1016/j.asjsur.2020.08.014 32912730

